# Kidney function in tenofovir disoproxil fumarate-based oral pre-exposure prophylaxis users: a systematic review and meta-analysis of published literature and a multi-country meta-analysis of individual participant data

**DOI:** 10.1016/S2352-3018(22)00004-2

**Published:** 2022-03-07

**Authors:** Robin Schaefer, Pedro Henrique Amparo da Costa Leite, Ronaldo Silva, Quarraisha Abdool Karim, Christopher Akolo, Carlos F Cáceres, Inês Dourado, Kimberly Green, Anita Hettema, Elske Hoornenborg, Smarajit Jana, Bernhard Kerschberger, Hally Mahler, Sindy Matse, Hamish McManus, Jean-Michel Molina, Sushena Reza-Paul, Iskandar Azwa, Maryam Shahmanesh, Doug Taylor, Hamid Vega-Ramirez, Valdiléa G Veloso, Rachel Baggaley, Shona Dalal

**Affiliations:** a Global HIV, Hepatitis and STIs Programmes, World Health Organization, Geneva, Switzerland; b Evandro Chagas National Institute of Infectious Diseases, Oswaldo Cruz Foundation, Rio de Janeiro, Brazil; c Department of Infectious-Tropical Diseases and Microbiology, IRCCS Sacro Cuore Don Calabria Hospital, Negrar, Italy; d CAPRISA, Nelson R Mandela School of Medicine; University of KwaZulu-Natal, Durban, South Africa; e FHI 360, Washington, DC, USA; f Centro de Investigación Interdisciplinaria en Sexualidad, SIDA y Sociedad, Universidad Cayetano Heredia, Lima, Peru; g Collective Health Institute, Federal University of Bahia, Brazil; h PATH Vietnam, Hanoi, Vietnam; i Clinton Health Access Initiative, Mbabane, Eswatini; j Center for Sexual Health, Department of Infectious Diseases, Public Health Service Amsterdam, Amsterdam, Netherlands; k Durbar Mahilla Samanwaya Committee, Kolkata, India; l Médecins Sans Frontières, Mbabane, Eswatini; m National AIDS Programme, Mbabane, Eswatini; n The Kirby Institute, University of New South Wales Sydney, Sydney, NSW, Australia; o Department of Infectious Diseases, St-Louis and Lariboisière Hospitals, University of Paris, INSERM U944, Paris, France; p Department of Community Health Sciences, University of Manitoba, Winnipeg, MB, Canada; q Ashodaya Samithi, Mysuru, India; r Infectious Diseases Unit, Faculty of Medicine, University of Malaya, Kuala Lumpur, Malaysia; s Africa Health Research Institute, KwaZulu-Natal, South Africa; t Institute for Global Health, University College London, London, UK; u FHI 360, Durham, NC, USA; v National Institute of Psychiatry Ramon de la Fuente Muñiz, Mexico City, Mexico

## Abstract

**Background:**

Previous WHO guidance on tenofovir disoproxil fumarate-based oral pre-exposure prophylaxis (PrEP) suggests measuring creatinine levels at PrEP initiation and regularly afterwards, which might represent barriers to PrEP implementation and uptake. We aimed to systematically review published literature on kidney toxicity among tenofovir disoproxil fumarate-based oral PrEP users and conducted an individual participant data meta-analysis (IPDMA) on kidney function among PrEP users in a global implementation project dataset.

**Methods:**

In this systematic review and meta-analysis we searched PubMed up to June 30, 2021, for randomised controlled trials (RCTs) or cohort studies that reported on graded kidney-related adverse events among oral PrEP users (tenofovir disoproxil fumarate-based PrEP alone or in combination with emtricitabine or lamivudine). We extracted summary data and conducted meta-analyses with random-effects models to estimate relative risks of grade 1 and higher and grade 2 and higher kidney-related adverse events, measured by elevated serum creatinine or decline in estimated creatinine clearance or estimated glomerular filtration rate. The IPDMA included (largely unpublished) individual participant data from 17 PrEP implementation projects and two RCTs. Estimated baseline creatinine clearance and creatinine clearance change after initiation were described by age, gender, and comorbidities. We used random-effects regressions to estimate the risk in decline of creatinine clearance to less than 60 mL/min.

**Findings:**

We identified 62 unique records and included 17 articles reporting on 11 RCTs with 13 523 participants in meta-analyses. PrEP use was associated with increased risk of grade 1 and higher kidney adverse events (pooled odds ratio [OR] 1·49, 95% CI 1·22–1·81; *I*^2^=25%) and grade 2 and higher events (OR 1·75, 0·68–4·49; *I*^2^=0%), although the grade 2 and higher association was not statistically significant and events were rare (13 out of 6764 in the intervention group *vs* six out of 6782 in the control group). The IPDMA included 18 676 individuals from 15 countries (1453 [7·8%] from RCTs) and 79 (0·42%) had a baseline estimated creatinine clearance of less than 60 mL/min (increasing proportions with increasing age). Longitudinal analyses included 14 368 PrEP users and 349 (2·43%) individuals had a decline to less than 60 mL/min creatinine clearance, with higher risks associated with increasing age and baseline creatinine clearance of 60·00–89·99 mL/min (adjusted hazard ratio [aHR] 8·49, 95% CI 6·44–11·20) and less than 60 mL/min (aHR 20·83, 12·83–33·82).

**Interpretation:**

RCTs suggest that risks of kidney-related adverse events among tenofovir disoproxil fumarate-based oral PrEP users are increased but generally mild and small. Our global PrEP user analysis found varying risks by age and baseline creatinine clearance. Kidney function screening and monitoring might focus on older individuals, those with baseline creatinine clearance of less than 90 mL/min, and those with kidney-related comorbidities. Less frequent or optional screening among younger individuals without kidney-related comorbidities may reduce barriers to PrEP implementation and use.

**Funding:**

Unitaid, Bill & Melinda Gates Foundation, WHO.

**Research in context**

**Evidence before this study**

We searched PubMed on June 30, 2021 with the following subject headings and keywords: (“pre-exposure prophylaxis”[MeSH Terms] OR “prep”[Title/Abstract]) AND ((((((((“serum”[Text Word] AND “creatinine”[Text Word]) OR (“renal”[Title/Abstract] AND “function”[Title/Abstract])) OR (“serum”[Title/Abstract] AND “creatinine”[Title/Abstract])) OR “chemistry”[Title/Abstract]) OR “glomerul*”[Title/Abstract]) OR “GFR”[Title/Abstract]) OR “MDRD”[Title/Abstract]) OR “Cockcroft”[Title/Abstract]), to identify studies that evaluated the effects of tenofovir disoproxil fumarate-based oral pre-exposure prophylaxis (PrEP) on kidney function. No restrictions were applied to publication year or language. 11 randomised controlled trials (RCTs) on tenofovir disoproxil fumarate-based oral PrEP were identified that provided sufficient information on kidney-related outcomes. Some trials, but not all, found a statistically increased risk in kidney adverse events among PrEP users. Previous meta-analyses found the risk of grade 1 and higher kidney adverse events to be statistically significantly higher in PrEP users, but these adverse events were generally mild and reversible. No meta-analysis of grade 2 and higher kidney adverse events was identified. Some studies found gradually increasing risk of kidney adverse events by age and by baseline creatinine clearance. Most PrEP trials did not evaluate factors associated with changes in kidney function over time. Few studies evaluated kidney function in PrEP users outside of clinical trial settings, and studies were limited to high-income settings.

**Added value of this study**

We conducted a systematic review and meta-analysis of 11 RCTs of oral PrEP and found that there was a statistically significantly increased risk of kidney-related adverse events in PrEP users, although these were rare and tended to be mild. Additionally, we analysed a global data set consisting of 17 PrEP implementation programmes and projects and two RCTs across 15 countries. Among 18 676 individuals screened for PrEP initiation, 0·42% had abnormal baseline estimated creatinine clearance (<60 mL/min), with increasing proportions with increasing age. Of 14 368 individuals who initiated PrEP and had follow-up information, 2·43% had a decline to less than 60 mL/min creatinine clearance, with higher risk associated with increasing age and a baseline creatinine clearance of less than 90 mL/min.

**Implications of all the available evidence**

Only a small fraction of people screened for tenofovir disoproxil fumarate-based oral PrEP initiation have a creatinine clearance of less than 60 mL/min, which would be a contraindication for oral PrEP, and kidney adverse evets among PrEP users are rare and generally non-progressive and reversible. Older individuals and those with a baseline creatinine clearance of less than 90 mL/min are at increased risk of clinically significant declines in creatinine clearance. Logistical challenges and costs associated with creatinine screening at and regularly after PrEP initiation might be barriers to PrEP service implementation and uptake. Less frequent or optional screening among younger individuals without kidney-related comorbidities might be appropriate.

## Introduction

In 2015, WHO recommended offering once-daily tenofovir disoproxil fumarate-based oral pre-exposure prophylaxis (PrEP) to people at substantial risk of HIV infection. Although tenofovir disoproxil fumarate-based oral PrEP is safe and generally well-tolerated, reviews found that some studies identified a statistically significant increase in the risk of kidney adverse events, while others did not.^1^, ^2^ These effects on kidney function were usually slight declines in estimated creatinine clearance or glomerular filtration rate (eGFR) that were non-progressive and reversible after discontinuation of PrEP.^3^, ^4^, ^5^ A meta-analysis found that severe kidney-related adverse events were extremely rare in clinical trials and not statistically significantly different between PrEP users and control individuals.^2^ Due to concerns of small risks of nephrotoxicity, WHO guidance,^6^ released in 2017, suggested measuring serum creatinine levels at the time of PrEP initiation to identify pre-existing kidney disease (indicated by estimated creatinine clearance of below 60 mL/min) and to conduct creatinine screening every 6 months thereafter (more frequent monitoring for people with kidney-related comorbidities and less frequent for people younger than 45 years, those with baseline estimated creatinine clearance of over 90 mL/min, and those weighing over 55 kg).

Since WHO's recommendation on oral PrEP, there has been a global increase in the adoption of PrEP into national guidelines and more widespread implementation of PrEP services.^7^ Nevertheless, logistical challenges and costs associated with creatinine screening at PrEP initiation and thereafter have been reported as barriers to PrEP implementation as well as uptake and effective use among users.^8^, ^9^, ^10^ With continuing roll-out and scale-up of PrEP services, efforts are underway to simplify PrEP service delivery to maximise uptake and effective use while minimising adverse effects, including the optimal monitoring procedures for kidney function. Identifying subgroups of individuals who might require less frequent kidney monitoring could reduce costs associated with PrEP services and remove barriers to access. The objectives of this study were to conduct a systematic review and meta-analysis of published data on kidney toxicity among PrEP users and an individual participant data meta-analysis in a global dataset of PrEP implementation projects and studies with wide geographical representation.

## Methods

### Search strategy and selection criteria for the systematic review of published literature

In this systematic review and meta-analysis we searched published literature on oral PrEP to identify randomised controlled trials (RCTs) and cohort studies with data on adverse events related to kidney function among PrEP users. We searched PubMed on June 30, 2021, using specific search terms (full list is in the appendix p1), and no restrictions were applied to publication year or language. Additional articles were identified by manually searching bibliographies of selected articles. Methods for study selection followed guidelines in the Preferred Reporting Items for Systematic Reviews and Meta-Analyses (PRISMA) statement.^11^ The review included studies published in a peer-reviewed journal evaluating tenofovir disoproxil fumarate-based PrEP alone or in combination with emtricitabine or lamivudine. Only cohort studies and randomised controlled trials were included. The outcomes of interest were graded kidney-related adverse events measured by elevated serum creatinine or decline in estimated creatinine clearance or eGFR. Most studies used the National Institutes of Health Division of AIDS definitions for kidney-related adverse events.^12^ PHAdCL implemented the search strategy. All studies identified for inclusion were additionally reviewed by RS. Conflict was resolved through consensus. Where this could not be reached, SD mediated. Extracted information included kidney-related outcomes, study design, sample size, and study drugs. Further details on the review methodology are in the appendix (p 1).

### Meta-analysis of the published literature

Meta-analyses estimated pooled relative risks of grade 1 and higher and grade 2 and higher kidney-related adverse events. Grade 1 and higher kidney-related adverse events included all serum creatinine elevations from 1·1 to 1·3 times the upper limit of typical levels. Grade 2 and higher events included serum creatinine elevations from 1·3 to 1·8 times the upper limit of typical or 1·3 to 1·5 times the participants' baseline value as well as creatinine clearance reductions to less than 90 mL/min or 10–30% reductions from participants' baseline values (appendix p 1). Random-effects models were used due to differences in clinical interventions and study populations. A continuity correction of 0·5 was added to studies with zero events in one study group. Studies with zero events in both study groups were excluded from pooled estimates in meta-analyses. Supplementary analyses were implemented using the Peto method for meta-analyses.^13^ Meta-analyses were implemented using R version 3.6.3.

### Individual participant data meta-analysis of global dataset

Data on PrEP use and kidney function were collected in a standardised format from 19 PrEP programmes and studies that responded to a call for data by WHO (most of these data have not been published before), including 17 implementation projects and two RCTs (FEM-PrEP; IPERGAY). For the placebo-controlled trials, only participants receiving active PrEP were included in the analysis. Details on collected data are shown in the appendix (p 9). The WHO Ethics Review Committee exempted this study from ethical review because de-identified secondary data were used and data contributors confirmed that applicable ethical principles and legal requirements were met in relation to the secondary use of these data.

Our key measure for kidney function was estimated creatinine clearance. Some PrEP projects only reported estimated creatinine clearance, not serum creatinine levels, so other measures of kidney function, such as eGFR estimated with the Chronic Kidney Disease Epidemiology Collaboration (CKD-EPI) equation, could not be used for all data. When creatinine clearance was not directly reported by the PrEP project, this was calculated based on serum creatinine and sex at birth, age, weight, and height using the Cockcroft-Gault equation.^14^ Estimated creatinine clearance stages were defined as 90 mL/min or more (normal kidney function), 60·00–89·99 mL/min (moderate kidney function), and less than 60 mL/min (abnormal kidney function).^12^ The primary outcome measure after PrEP initiation was a deterioration to a clinically significant estimated creatinine clearance of less than 60 mL/min (referred to as a clinically significant decline), indicating onset of kidney impairment.

Individuals who were screened for PrEP and had a creatinine measurement were included in the baseline analysis regardless of whether they initiated PrEP. Proportions of individuals with different creatinine clearance stages were described by age (15–19 years, 20–24 years, 25–29 years, 30–39 years, 40–49 years, and older than 50 years), gender (cisgender male or female, transgender male or transgender female, and non-binary), and known comorbidity potentially associated with kidney function (diabetes, dyslipidaemia, and hypertension).

Individuals who initiated PrEP and had at least one follow-up creatinine measurement were included in longitudinal analyses. Individuals were censored after a decline in estimated creatinine clearance to less than 60 mL/min or at the last recorded creatinine measurement. Proportions and unadjusted survival curves of clinically significant declines (<60 mL/min creatinine clearance) were calculated by time of follow-up (<3 months, 3–6 months, 6–12 months, and >12 months), baseline age, gender, known comorbidity, and baseline creatinine clearance stage. Random-effects regressions for time to event data based on a Cox proportional hazards model were fitted with age group, gender, and baseline creatinine clearance stage as fixed effects, and PrEP study or project as random effects. Analyses were implemented using all data and separately for low-income and middle-income countries (LMICs) and high-income countries (HICs). Proportional hazards assumptions of Cox models were checked using graphical methods. Missing data for covariates were rare. No data were imputed and denominators for different analyses represent available data. Analyses were implemented in SAS version 9.4.

### Role of the funding source

The funders of the study had no role in study design, data collection, data analysis, data interpretation, or writing of the report.

## Results

**Figure 1:**
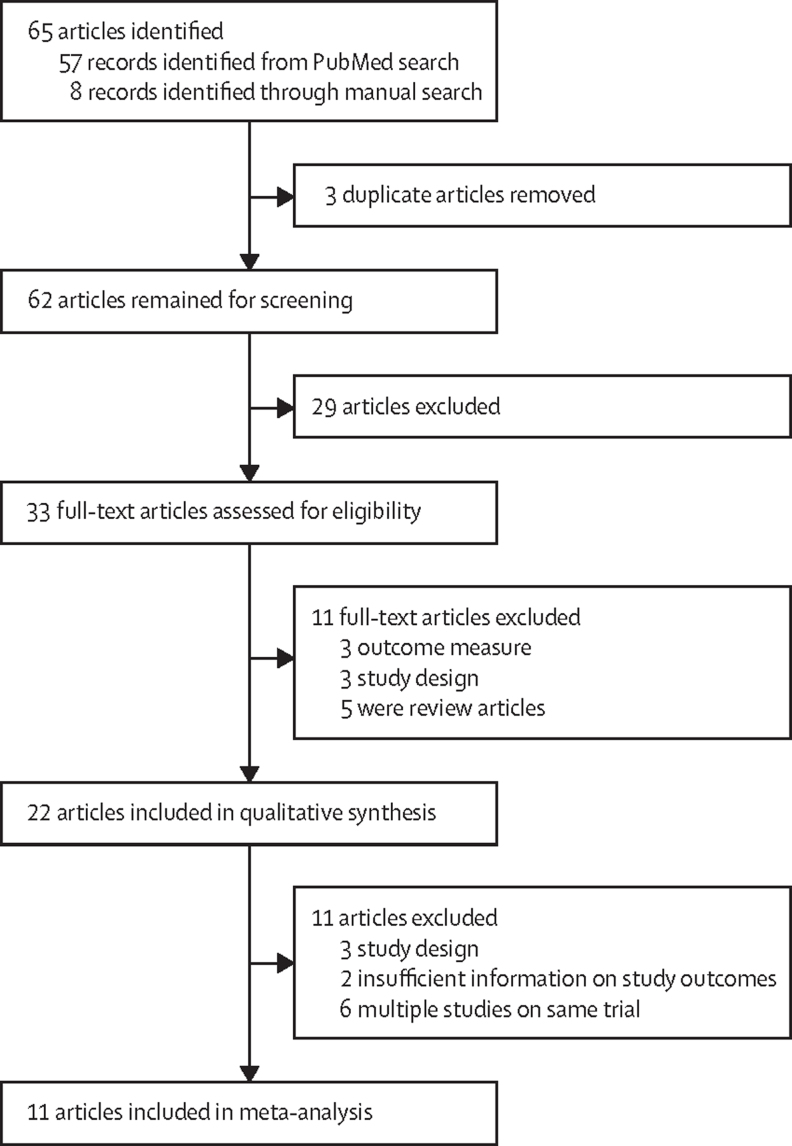
Study selection from the systematic search of published literature on kidney-related adverse events in tenofovir disoproxil fumarate-based oral PrEP users PrEP=pre-exposure prophylaxis.

**Figure 2:**
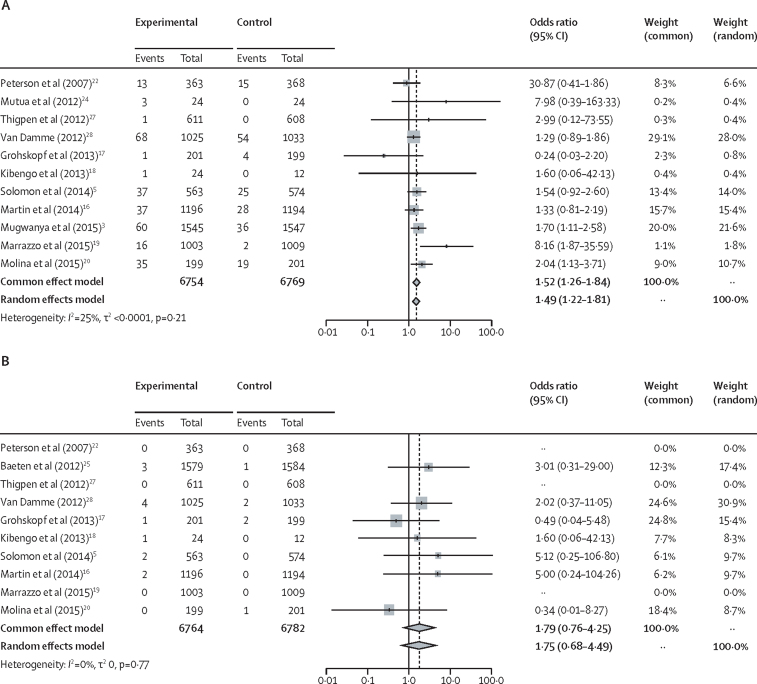
Meta-analysis of kidney-related adverse events in published randomised controlled trials on tenofovir disoproxil fumarate-based oral pre-exposure prophylaxis Forest plots showing risks among tenofovir disoproxil fumarate-based oral PrEP users for grade 1 and higher kidney-adverse events (A) and grade 2 and higher kidney-related adverse events (B). Most studies evaluated kidney-related adverse events with elevations in serum creatinine. Kidney-related adverse events were graded according to the National Institutes of Health, Division of AIDS^12^ definitions and further details are in the appendix (p 1). PrEP=pre-exposure prophylaxis.

The literature search identified 62 unique records, of which 22 were included in the review (figure 1). 17 articles reported on 11 different RCTs were included in the meta-analyses.^3^, ^5^, ^15^, ^16^, ^17^, ^18^, ^19^, ^20^, ^21^, ^22^, ^23^, ^24^, ^25^, ^26^, ^27^, ^28^, ^29^. Most studies defined kidney-related adverse events as elevations in serum creatinine levels (table 1). In most individual studies, risks of kidney adverse events did not differ significantly between PrEP users and control groups. In the pooled meta-analysis, PrEP use was associated with a significantly increased risk of grade 1 and higher adverse events (13 523 study participants; odds ratio [OR] 1·49, 95% CI 1·22–1·81; *I*^2^=25%, p=0·21; figure 2A). There was an increased risk of grade 2 and higher events among PrEP users (13 546 study participants; OR 1·75, 0·68–4·49; *I*^2^=0%, p=0·77; figure 2B), but these were rare (13 grade 2 and higher events among 6764 PrEP users *vs* six among 6782 control individuals). Results using the Peto method were similar (grade 1 and higher OR 1·52, 1·26–1·84; grade 2 and higher OR 2·04, 0·83–5·02; appendix p 6). Two additional RCTs were identified that were not included in the primary meta-analyses because insufficient information on kidney function measures were provided.^30^, ^31^ Sensitivity analyses included these studies and found similar risks for grade 1 and higher (OR 1·53, 1·20–1·94) and grade 2 and higher kidney-related adverse events (OR 1·89, 0·79–4·52; appendix pp 7, 8). Detailed results of the whole systematic review and meta-analyses are in the appendix (pp 2–8).

**Table 1:** Summary of randomised controlled trials on effects of tenofovir disoproxil fumarate-based oral PrEP on kidney function identified in the systematic review of the published literature and included in the meta-analysis

	**Study name**	**Study countries**	**Population**	**Study drug**	**Enrolment**	**Number of participants**	**Serum creatinine measure frequency**	Estimated creatinine clearance or estimated glomerular filtration rate*
Peterson et al (2007)^22^	West African Safety Study	Ghana, Cameroon†	Cisgender women	Tenofovir disoproxil fumarate (oral)	June, 2004, to March, 2006	859	Enrolment; and months 1, 3, 6, 9, and 12	None
Grant et al (2010);^23^ Solomon et al (2014)^5^	iPrEx	Brazil, Ecuador, Peru, South Africa, Thailand, USA	Cisgender men who have sex with men and transgender women	Tenofovir disoproxil fumarate (oral) plus emtricitabine (oral)	July, 2007, to December, 2009	2499‡	Enrolment; weeks 4, 8, 12, 16, and 24; and every 12 weeks thereafter	Cockcroft-Gault equation
Mutua et al (2012)^24^	IAVI-Kenya	Kenya	Cisgender men who have sex with men and female sex workers	Tenofovir disoproxil fumarate (oral) plus emtricitabine (oral)	October to December, 2009	36	Monthly	Cockcroft-Gault equation
Baeten et al (2012);^25^ Mugwanya et al (2015);^3^ Mugwanya et al (2016)^26^	Partners PrEP	Kenya, Uganda	Serodifferent couples	Tenofovir disoproxil fumarate (oral); and tenofovir disoproxil fumarate (oral) plus emtricitabine (oral)	July, 2008, to November, 2010	4640	Baseline; month 1; and every 3 months thereafter	Cockcroft-Gault equation, CKD-EPI, and markers of proximal tubular dysfunction
Thigpen et al (2012)^27^	TDF2	Botswana	Cisgender men who have sex with men and cisgender women	Tenofovir disoproxil fumarate (oral) plus emtricitabine (oral)	March, 2007, to October, 2009	1219	Monthly	None
Van Damme et al (2012);^28^ Mandala et al (2014)^29^	FEM-PrEP	Kenya, South Africa, Tanzania	Cisgender women	Tenofovir disoproxil fumarate (oral) plus emtricitabine (oral)	June, 2009, to April, 2011	2058	Enrolment; weeks 4, 12, 24, 36, 52, and 56; and when clinically indicated	None
Choopanya et al (2013);^15^ Martin et al (2014)^16^	Bangkok Tenofovir	Thailand	People who inject drugs	Tenofovir disoproxil fumarate (oral)	June, 2005, to July, 2010	2413	Enrolment; months 1, 2, and 3; and every 3 months thereafter	Cockcroft-Gault equation, MDRD, and CKD-EPI
Grohskopf et al (2013)^17^	US Safety	USA	Cisgender men who have sex with men	Tenofovir disoproxil fumarate (oral)	January, 2005, to July, 2007	400	Enrolment; and months 1, 3, 6, 9, 12, 15, 18, 21, and 24	None
Kibengo et al (2013)^18^	IAVI-Uganda	Uganda	Serodifferent couples	Tenofovir disoproxil fumarate (oral) plus emtricitabine (oral)	October, 2009, to March, 2010	36	Enrolment and monthly visits	Cockcroft-Gault equation
Marrazzo et al (2015)^19^	VOICE trial (MTN-003)	South Africa, Uganda, Zimbabwe	Cisgender women	Tenofovir disoproxil fumarate (oral) and tenofovir disoproxil fumarate plus emtricitabine (oral and vaginal gel§)	September, 2009, to June, 2011	5029	Monthly	None
Molina et al (2015);^20^ Liegeon et al (2020)^21^	ANRS-IPERGAY	Canada, France	Cisgender men who have sex with men and transgender women	Tenofovir disoproxil fumarate (oral) plus emtricitabine (oral)	February, 2012, to January, 2015; on demand PrEP 2014–15	400	Enrolment; week 4; and every 8 weeks thereafter	Cockcroft-Gault equation and CKD-EPI

CKD-EPI=chronic kidney disease epidemiology collaboration equation. MDRD=modification of diet in renal disease equation. PrEP=pre-exposure prophylaxis.

* Methods to estimate creatinine clearance refers to the Cockcroft-Gault equation and estimated glomerular filtration rate was estimated either with the CKD-EPI equation or the MDRD equation.

† Data from Nigeria were not included in analyses of renal function.

‡ The sample size for the analysis by Solomon et al (2014) for the iPrEx study was 1137.

§ Only the data from oral PrEP participants were included in the meta-analysis.

The global dataset of PrEP users included 18 676 individuals screened for PrEP initiation across 15 countries (table 2), of which 7382 (39·5%) were from HICs (Australia, France, and the Netherlands), 6023 (32·2%) from the Americas (Brazil, Mexico, Peru), 3304 (17·7%) from sub-Saharan Africa (Eswatini, Kenya, Malawi, South Africa, and Tanzania), and 1967 (10·5%) from Asia (India, Malaysia, Nepal, and Vietnam). The two included RCTs contributed 1453 (7·8%) of individuals screened for PrEP. Half of the individuals were younger than 30 years (9040 [48·4%] of 18 629; median 30 years, IQR 24–37), and 14 194 (76·0%) of 18  674 identified as cisgender male, 4023 (21·5%) as cisgender female, and 457 (2·4%) as non-binary or transgender.

**Table 2:** Background information on data on creatinine screening and kidney-related adverse events among PrEP users by PrEP study or programme included in the individual participant data meta-analysis of data from 15 countries

		**Country**	**Median age, years**	**Cisgender**		**Transgender**		**Non-binary**	Analysed sample*			
				Male	Female	Male	Female		Baseline	Follow-up	Percentage of participants with follow-up of 6 or more months	Median follow-up time, months
Complete dataset		..	30 (24–37)	14 238	4034	35	399	28	18 676	14 384	71·9%	10 (6–15)
Americas region												
	ImPrEP Mexico	Mexico	30 (26–35)	501	5	1	12	0	487	265	64·9%	6 (6–8)
	ImPrEP Peru	Peru	27 (23–34)	1296	25	0	174	0	1490	473	92·6%	8 (7–11)
	ImPrEP Brazil	Brazil	29 (24–35)	3550	26	5	145	0	3722	2343	78·2%	7 (6–12)
	PrEP1519	Brazil	18 (18–19)	298	0	0	29	0	324	266	29·3%	3 (1–6)
African region												
	FHI360 Eswatini	Eswatini	30 (25–37)	64	38	0	0	0	100	53	11·3%	3 (1–4)
	MOH Eswatini†	Eswatini	28 22–37)	51	123	0	0	0	173	13	53·9%	7 (5–9)
	MSF Eswatini	Eswatini	27 (22–33)	103	379	0	0	0	474	144	43·1%	6 (3–9)
	FHI360 Malawi	Malawi	23 (20–27)	0	383	0	0	0	383	89	6·74%	2 (1–4)
	CAPRISA	South Africa	26 (22–33)	339	495	0	0	0	834	552	33·9%	4 (3–9)
	AHRI	South Africa	23 (20–25)	164	151	0	0	0	315	138	19·6%	1 (1–5)
	FEM-PrEP‡	South Africa, Kenya, Tanzania	23 (20–27)	0	1025	0	0	0	1025	1020	78·2%	9 (6–13)
European region												
	IPERGAY§	France	34 (29–42)	427	0	2	0	0	428	426	91·8%	25 (19–34)
	AMPrEP	Netherlands	40 (32–48)	374	0	0	2	0	376	367	94·8%	34 (25–36)
South-East Asia region												
	MyHome Clinic†	Vietnam	28 (25–31)	372	36	0	0	0	410	282	32·6%	6 (6–12)
	FHI360 Nepal	Nepal	24 (20–31)	49	23	0	23	0	94	60	3·33%	3 (3–4)
	DMSC	India	28 (25–35)	0	675	0	0	0	672	646	94·4%	15 (15–15)
	Ashodaya	India	35 (30–40)	0	647	0	0	0	647	660	91·2%	16 (15–16)
Western Pacific region												
	MyPrEP	Malaysia	28 (25–34)	144	0	0	0	0	144	139	97·1%	12 (11–12)
	EPIC NSW	Australia	34 (28–43)	6506	3	27	14	28	6578	6448	70.5%	12 (6–20)

Data are median (IQR), n, or %. Regions refer to WHO regions. PrEP=pre-exposure prophylaxis.

* Individuals were included in analyses of the baseline data if they had a creatinine measurement. The numbers of sample sizes by gender might not add up to the analysed baseline sample due to missing creatinine information. Data on follow-up time was restricted to those included in the longitudinal analysis and refers to the time from PrEP initiation to censoring. Follow-up analyses included individuals with at least one follow-up creatinine measurement after PrEP initiation.

† Data were reported for sex only and individuals were classified as cisgender male or cisgender female.

‡ The FEM-PrEP randomised placebo-controlled trial was implemented in Kenya, South Africa, and Tanzania. Most individuals were from Kenyan (34·8%) or South African (62·3%) study sites. Only individuals in the active PrEP study arm of the trial were included in the analysis.

§ The IPERGAY study was a randomised placebo-controlled trial to evaluate on-demand oral PrEP use. Only individuals in the active PrEP study arm of the trial were included in the analysis. Individuals using oral PrEP in the open-label extension of the trial were also included in the analysis.

At baseline, 79 (0·42%) of 18 676 individuals screened for PrEP had an abnormal estimated creatinine clearance of less than 60 mL/min, and 4121 (77·5%) had estimated creatinine clearance of 90 mL/min or more. Proportions of individuals with less than 60 mL/min baseline creatinine clearance increased with age (from one [0·09%] among 15–19 year olds to 23 [1·83%] among those older than 50 years) and were higher among cisgender females than cisgender males (34 [0·85%] of 4023 *vs* 45 [0·32%] of 14 194; appendix p 10). Sample sizes for transgender and non-binary individuals were small and no individual had abnormal creatinine clearance (estimated clearance of less than 60 mL/min). Data on comorbidities were limited; among 110 individuals with known kidney-related comorbidities, three (2·7%) had abnormal creatinine clearance.

Data on 14 368 individuals were included in the longitudinal analysis (7241 [50·4%] from HICs; 1446 [10·0%] from RCTs). The median follow-up time was 10 months (IQR 6–15; range 0–51), with marked variation across projects and studies (table 2). Follow-up information for at least 6 months after PrEP initiation was available for 10 330 [71·9%] of 14 368; 5934 [41·3%] of 14 368 were followed up for at least 12 months. At 18 months of follow-up, most data (2559 of 2601 individuals) were from HICs. A clinically significant decline in creatinine clearance to less than 60 mL/min was observed in 349 (2·43%) of 14 368 individuals. Most declines (263 [75·4%]) occurred within 12 months of initiation; 173 [49·6%] occurred within 6 months (table 3). Proportions of individuals with clinically significant declines decreased with increasing time on PrEP (table 3). Among the 349 individuals with a less than 60 mL/min decline, 263 (75·4%) had another follow-up measurement, and of these 217 (82·8%) returned to a creatinine clearance of 60 mL/min or more at the subsequent measurement. 80 individuals had at least two declines to less than 60 mL/min (median time to second decline: 8 months).

**Table 3:** Clinically significant declines in estimated creatinine clearance to <60 mL/min by time of the creatinine measurement after PrEP initiation between high-income and low-income and middle-income countries in 15 countries

	**Low-income and middle-income countries**		**High-income countries**	
	<60 mL/min decline	Cumulative percentage of all declines to <60 mL/min	<60 mL/min decline	Cumulative percentage of all declines to <60 mL/min
0–1 months after PrEP initiation	36/384 (9·4%)	20·3%	18/88 (20·5%)	10·5%
2–3 months after PrEP initiation	22/703 (3·1%)	32·8%	33/922 (3·6%)	29·7%
4–6 months after PrEP initiation	38/1502 (2·5%)	54·2%	26/1133 (2·3%)	44·8%
7–12 months after PrEP initiation	56/2643 (2·1%)	85·9%	34/1419 (2·4%)	64·5%
>12 months after PrEP initiation	25/1898 (1·3%)	100·0%	61/3676 (1·7%)	100·0%

Data are n/N (%) or %. PrEP=pre-exposure prophylaxis.

**Figure 3:**
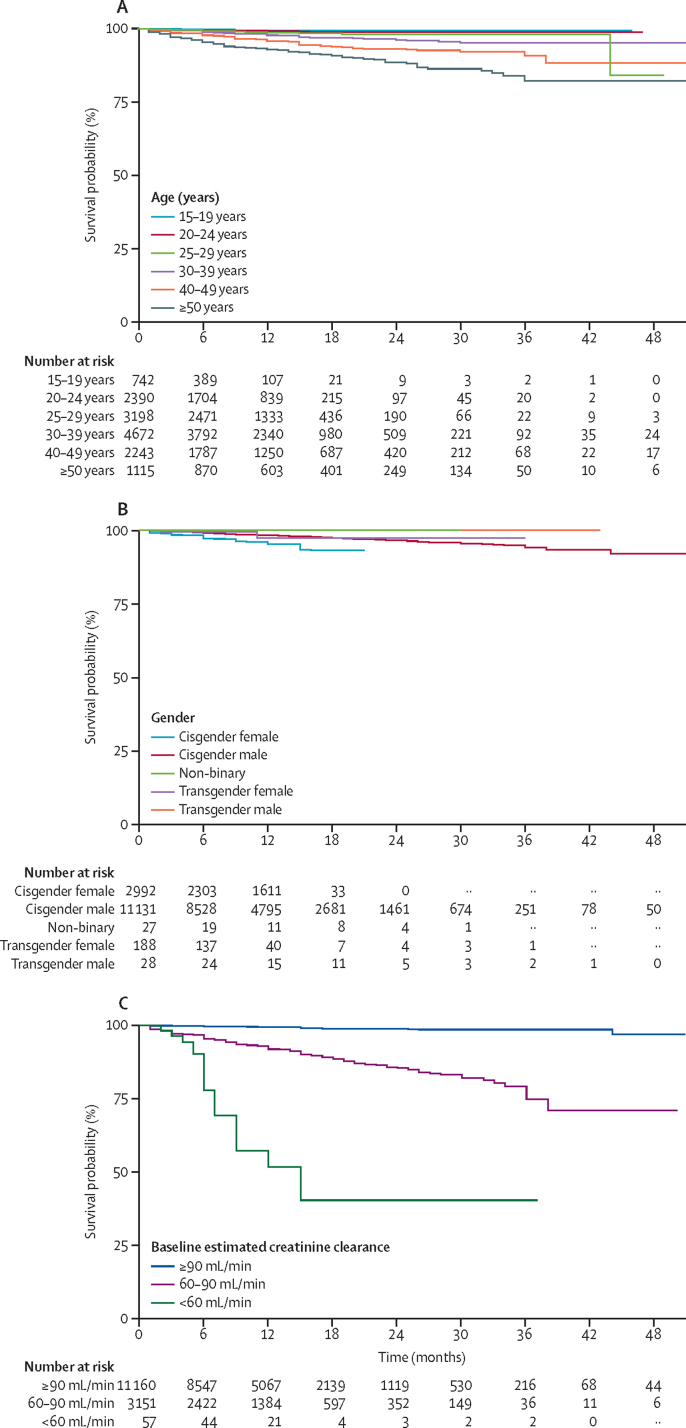
Cumulative probability of maintaining an estimated creatinine clearance of 60 mL/min or more over time after PrEP initiation in PrEP users from 15 countries by age group (A), gender (B), and baseline estimated creatinine clearance (C). Numbers indicate individuals at risk of a clinically significant decline in estimated creatinine clearance to less than 60 mL/min by different times of follow-up. PrEP=pre-exposure prophylaxis.

Proportions of individuals with a decline in estimated creatinine clearance to less than 60 mL/min increased by age (figure 3). The median age of those who had such a decline was 40 years (IQR 34–51). After controlling for gender and baseline creatinine clearance stage, those older than 50 years had a significantly higher risk of a clinically significant decline in creatinine clearance (adjusted hazard ratio [aHR] 6·05, 95% CI 1·41–26·0; appendix p 11). Of the 349 individuals who had a clinically significant decline, 254 (72·8%) had a creatinine clearance of 60·00–89·99 mL/min at baseline and 79 (22·6%) had a creatinine clearance of 60·00–69·99 mL/min; 7·16% had a baseline creatinine clearance of less than 60 mL/min (those with a baseline creatinine clearance of <60 mL/min were only counted as a decline it they had ≥60 mL/min at a subsequent visit). After controlling for age and gender, there was an eight-fold increase in the risk of a clinically significant decline among those with a baseline estimated creatinine clearance of 60·00–89·99 mL/min (aHR 8·49, 95% CI 6·44–11·20) and a 20-fold increased risk among those with less than 60 mL/min than those with a baseline of 90 mL/min or more (aHR 20·83, 12·83–33·82; appendix p 11). Although not statistically significant, cisgender females tended towards an increased risk of a clinically significant decline (aHR 2·43, 0·98–6·00). A decline to less than 60 mL/min was reported in 136 (4·55%) of 2992 cisgender females, compared with 211 (1·90%) of 11 131 cisgender males. However, 90% of the cisgender women with a creatinine clearance of less than 60 mL/min (n=123) came from two projects in India (Ashodaya Samithi) and Eswatini (FHI 360) that contributed 662 (22·1%) of 2992 women included in the longitudinal analysis. When excluding these two projects, no difference in risk between cisgender women and men was observed (aHR 1·12, 0·38–3·32), and effects by age and baseline creatinine clearance remained the same (appendix p 12). The effect was also reduced when limiting analyses to LMICs (aHR 1·64, 0·64–4·19; appendix p 11). There was no risk difference between cisgender and transgender or non-binary individuals, although data were scarce (appendix p 11). Data on comorbidities were too scarce to be included in regressions.

## Discussion

Our study suggests that few people who initiate tenofovir disoproxil fumarate-based oral PrEP experience clinically significant kidney impairment. The systematic review of published literature showed an increased risk of grade 1 and higher and grade 2 and higher events among PrEP users, but adverse events, particularly grade 2 and higher events, were rare, non-progressive, and resolved with PrEP discontinuation. This finding was similarly reported in previous reviews that did not distinguish between grade 1 and higher and grade 2 and higher events.^1^, ^2^ Less than 1% of people screened for PrEP initiation across 15 countries had an estimated creatinine clearance of less than 60 mL/min and fewer than 3% had a clinically significant decline in creatinine clearance to less than 60 mL/min after initiation. Moreover, more than 80% of individuals with creatinine clearance of less than 60 mL/min and available data returned to typical levels at their next measurement.

Our analysis of 19 PrEP projects and studies highlights that risks of having a clinically relevant decline in estimated creatinine clearance increase with age, with highest risks among those older than 50 years. This finding was similarly found in other studies,^16^, ^32^, ^33^, ^34^ although the ANRS-IPERGAY study of event-driven PrEP found no association between age and decline in eGFR after initiation.^21^ Risks of declines in estimated creatinine clearance were low for all genders, but we found slightly higher risks among cisgender women than cisgender men. Some studies among people living with HIV receiving tenofovir disoproxil fumarate-based antiretroviral therapy found risks of kidney impairment to be low but modestly higher in women,^35^, ^36^ and others found higher risks in men^37^, ^38^ or no association by sex.^39^ In our study, the higher risk among cisgender women might have been an artefact of the PrEP dosing regimen, because men in our dataset were more likely to live in HICs where event-driven PrEP was offered. Previous studies have found a dose-response relationship between tenofovir disoproxil fumarate exposure and declines in creatinine clearance,^21^, ^32^ suggesting that lower tenofovir disoproxil fumarate exposure of event-driven PrEP might have a comparatively better kidney safety profile. In our study, it was not possible to differentiate between PrEP dosing regimens; however, event-driven PrEP was offered in all HIC projects (although uptake in Australia was low) and was not offered in LMIC studies and programmes, so most individuals from LMICs in our study probably used daily PrEP. If men were more likely to be on event-driven PrEP, and event-driven PrEP was associated with better kidney safety, this would bias results towards higher risk among women; however, adherence to oral PrEP might be low and, outside of trial settings, often represent intermittent use during periods of risk, which would reduce tenofovir disoproxil fumarate exposure even among those using daily PrEP. The increased risk among cisgender women was reduced when restricting the analysis to LMICs, and no difference was observed when removing data from two projects with large proportions of cisgender women having clinically significant declines (representing 22% of women but 90% of declines). It is unclear why those two projects had disproportionally high proportions of women with declines in creatinine clearance and might reflect a high prevalence of risk factors such as comorbidities or random effects (one project had a small sample of only 53 individuals).

Data on adherence to oral PrEP were not available and a dose-response relationship between PrEP use and effects on kidney function could not be evaluated. Moreover, effects of oral PrEP use on kidney function might only present themselves after a prolonged period of use. In our study, sizable numbers of individuals were lost to follow-up shortly after initiation, although nearly 6000 participants had at least 12 months of follow-up. There was a trend of decreasing proportions of individuals with declines in creatinine clearance with increasing time of follow-up, with similar patterns in LMICs and HICs. However, this trend might be a form of survival bias, with individuals having adverse events discontinuing PrEP shortly after initiation. The loss-to-follow-up after PrEP initiation limits evaluations of longer-term effects of oral PrEP use on kidney function. Nevertheless, this loss-to-follow-up reflects patterns of PrEP use observed in many settings, with PrEP users commonly discontinuing shortly after initiation.^40^ Unlike antiretroviral therapy, oral PrEP can be used intermittently, with individuals going through cycles of starting and stopping PrEP depending on risks of exposure.^41^, ^42^, ^43^ Individuals might also switch between daily and event-driven PrEP.^44^ Using PrEP during periods of risk reduces overall tenofovir disoproxil fumarate exposure and thus risks of kidney-related adverse events.

Although individuals with pre-existing kidney conditions were not eligible for clinical trials on PrEP, our global data consisted largely of PrEP implementation projects, including over 18 000 individuals screened for PrEP, and few individuals had an estimated creatinine clearance of less than 60 mL/min (a contraindication for oral PrEP). Baseline estimated creatinine clearance decreased with age, which is consistent with other studies.^21^, ^32^ After controlling for age and gender, baseline creatinine clearance was the strongest determinant of risk of a creatinine clearance of less than 60 mL/min after PrEP initiation, which has also been reported elsewhere.^32^ Most individuals who had a clinically significant decline had a baseline creatinine clearance of 60·00–89·99 mL/min.

Estimated creatinine clearance was used to measure kidney function as it was available for all data. Urinary inulin clearance measurement is the gold standard for measuring GFR but difficult to implement routinely.^45^ Alternative measures, such as eGFR estimated with the Modification of Diet in Renal Disease (MDRD) or CKD-EPI equations, were unavailable or only available for a sub-set of data and could not be used to validate analyses with estimated creatinine clearance. The Cockcroft-Gault equation for estimating creatinine clearance was derived from a small sample of Canadian men and MDRD-estimated or CKD-EPI-estimated eGFR is considered a more accurate measure of GFR than Cockcroft-Gault-estimated creatinine clearance.^45^, ^46^, ^47^ However, these alternative equations include race and have only been validated in particular populations,^48^ although including race into eGFR equations has been called into question.^49^ Creatinine clearance estimated with the Cockcroft-Gault equation depends on sex, age, weight, and serum creatinine levels. Misclassification errors in any variable could have resulted in inaccurate creatinine clearance estimates but such errors were probably random and unlikely to introduce bias. Serum creatinine levels, however, can vary considerably within short time periods, are influenced by factors such as diet and posture,^50^, ^51^ and might be affected by a lack of standardised measurement.^52^ Therefore, levels of baseline estimated creatinine clearance of less than 60 mL/min and incidence of clinically significant declines after PrEP initiation could have been overestimated in this study, particularly as single measurements of creatinine clearance were used. Moreover, about 23% of those who had a decline to less than 60 mL/min had a baseline creatinine clearance of 60·00–69·99 mL/min, so actual declines in creatinine clearance could have been small and deemed clinically insignificant, further underscoring that clinically significant deteriorations in kidney functions could have been overestimated. In our study, more than 80% of individuals with a less than 60 mL/min decline in creatinine clearance had typical creatinine clearance at the subsequent measurement. Although it was not clear whether this repeat measurement was after PrEP discontinuation, the fact that 80% of individuals had a normal measure at next visit underscores the need to repeat kidney function measurements before excluding individuals from PrEP services. Estimated creatinine clearance also tends to underestimate GFR among older individuals,^45^ so proportions of older individuals with clinically significant kidney impairment might be lower than indicated by creatinine clearance.

Although our study included data from 15 countries, some countries were overrepresented in the data, notably Australia and Brazil. Given that PrEP users in these data were overwhelmingly cisgender males, cisgender females were comparatively underrepresented. Nevertheless, we included over 4000 cisgender females screened for PrEP. The random-effects regression model accounted for PrEP study or programme. Sensitivity analyses, excluding data from HICs, also found similar patterns of risks associated with age and baseline creatinine clearance. Risks of transgender and non-binary individuals could not be evaluated due to too few data. Effects of comorbidities or drugs associated with kidney function could also not be evaluated due to limited sample sizes. Effects of body-mass index could not be evaluated as creatinine clearance was estimated with the Cockcroft-Gault equation, which includes weight as a variable, and too few datasets included information on eGFR. Effects of ethnicity could not be evaluated due to inconsistent and incomplete reporting across datasets and countries.

In conclusion, our review of published literature and analysis of data from 17 PrEP implementation projects and two RCTs shows that only a small fraction of individuals screened for PrEP initiation have a low estimated creatinine clearance that would be a contraindication for PrEP (particularly among those younger than 30 years), and very few PrEP users had a clinically significant decline in creatinine clearance after initiation. Older age and baseline estimated creatinine clearance of less than 90 mL/min were associated with clinically significant declines in creatinine clearance. Although some programmes might choose to screen all PrEP users, creatinine screening and monitoring are associated with costs for health systems and place additional burden on PrEP users. Less frequent or optional creatinine screening among individuals younger than 30 years without kidney-related comorbidities might be appropriate given the low risks in this population. Moreover, PrEP delivery could be simplified with less frequent monitoring where resources allow, such as once within 1–3 months after PrEP initiation, for older individuals without comorbidities, although risks remain low even in the 30–49 year age group, particularly those aged 30–39 years. For individuals older than 50 years, those with low baseline creatinine clearance, and those with comorbidities, more regular monitoring might be required. A more focused monitoring schedule has been suggested by WHO in the updated PrEP guidance,^53^ which might aid in reducing barriers to the implementation and scale-up of oral PrEP services.

## Data sharing

Data used in the individual participant data meta-analysis were contributed from 19 different PrEP studies and programmes that shared data with WHO under specific conditions. Therefore, the complete dataset used in this study cannot be shared. Requests for individual datasets that were contributed to this study are considered on a case-to-case basis. Proposals should be sent to schaeferr@who.int and will be directed to the principal investigators of the contributing study or programme.

## Declaration of interests

J-MM declares that his institution has received grants from Gilead Sciences; receiving consulting fees from Gilead Sciences, Merck & Co, and ViiV Healthcare; and payments for expert testimony from Merck & Co, unrelated to the present work. KG declares receipt of travel grants by IAS and WHO to attend meetings unrelated to the present work.

All other authors declare no competing interests.
