# Supplementary material for: Kidney function in tenofovir disoproxil fumarate-based oral pre-exposure prophylaxis users: a systematic review and meta-analysis of published literature and a multi-country meta-analysis of individual participant data
Source: Lancet HIV. 2022 Mar 7;9(4):e242–53. doi: 10.1016/S2352-3018(22)00004-2 (PMC8964504; doi:10.1016/S2352-3018(22)00004-2)
Supplement: Supplementary appendix [file mmc1.pdf]

THE LANCET
HIV

Supplementary appendix

This appendix formed part of the original submission and has been peer reviewed. We post it as supplied by the authors.

Supplement to: Schaefer R, Amparo da Costa Leite P H, Silva R, et al. Kidney function in tenofovir disoproxil fumarate-based oral pre-exposure prophylaxis users: a systematic review and meta-analysis of published literature and a multi-country meta-analysis of individual participant data. *Lancet HIV* 2022; published online March 7. https://doi.org/10.1016/S2352-3018(22)00004-2.

Kidney function in tenofovir disoproxil fumarate-based oral pre-exposure prophylaxis users: a systematic review and meta-analysis of published literature and a multi-country meta-analysis of individual participant data

Schaefer et al.

Appendix

Systematic literature review and meta-analysis: Search strategy, data extraction, and analysis

The PubMed online database was searched with the following medical subject headings and keywords (last search: 30 June 2021): ("pre-exposure prophylaxis"[MeSH Terms] OR "prep"[Title/Abstract]) AND ((((((("serum"[Text Word] AND "creatinine"[Text Word]) OR ("renal"[Title/Abstract] AND "function"[Title/Abstract])) OR ("serum"[Title/Abstract] AND "creatinine"[Title/Abstract])) OR "chemistry"[Title/Abstract]) OR "glomerul*"[Title/Abstract]) OR "GFR"[Title/Abstract]) OR "MDRD"[Title/Abstract]) OR "Cockcroft"[Title/Abstract]). This search was supplemented by manually searching bibliographies of selected articles and relevant review articles. Studies were included if they were published in a peer-reviewed journal evaluating TDF-based PrEP, alone or in combination with FTC, and reported on kidney-related outcomes. The search strategy was implemented by the primary reviewer. All studies identified for inclusion were reviewed by a secondary reviewer. Relevant information was extracted from studies by the primary reviewer. This included information on kidney-related outcomes, study design, sample size, and study drugs. Data for the risk of bias assessment (see below) was extracted by the secondary reviewer. Data from randomised controlled trials were included in the meta-analysis and forest plots.

Systematic literature review and meta-analysis: Definition of outcomes for meta-analysis

Meta-analyses were conducted to estimate pooled relative risks of grade 1+ and grade 2+ kidney-related adverse events. These Grade 1+ and Grade 2+ adverse events were defined as per National Institutes of Health, Division of AIDS (DAIDS). See Table S1 below.

Table S1: Kidney-related adverse events as defined by the National Institutes of Health, Division of AIDS.

	Grade 1 (mild)	Grade 2 (moderate)	Grade 3 (severe)	Grade 4 (potentially life-threatening)
**Serum creatinine**	1.1 to 1.3 x ULN	> 1.3 to 1.8 x ULN OR Increase to 1.3 to < 1.5 x participant's baseline	> 1.8 to < 3.5 x ULN OR Increase to 1.5 to < 2.0 x participant's baseline	≥ 3.5 x ULN OR Increase of ≥ 2.0 x participant's baseline
**Creatinine clearance**	NA	< 90 to 60 ml/min or ml/min/1.73 m2 OR 10 to < 30% decrease from participant's baseline	< 60 to 30 ml/min or ml/min/1.73 m2 OR 30 to < 50% decrease from participant's baseline	< 30 ml/min or ml/min/1.73 m2 OR ≥ 50% decrease from participant's baseline or dialysis needed

ULN: Upper limited of normal; NA: Not applicable

Systematic literature review and meta-analysis: Risk of bias assessment
(See references in the main article and at the end of this appendix.)

17 articles on 11 randomised controlled trials (RCTs) were included in the primary meta-analyses. Interventions differed between studies, with four studies assessing the use of TDF alone (Peterson et al. 2007; Choopanya et al., 2013; Groshkopf et al., 2013; Martin et al., 2014); eight studies, the combination with FTC (Grant et al., 2010; Thigpen et al., 2012; Van Damme et al., 2021; Mandala et al., 2014; Molina et al., 2015; Lidgeon et al., 2020; Mutua et al., 2012; Kibengo et al., 2013); and four studies, both interventions (Baeten et al., 2021; Mugwanya et al., 2015; Marrazo et al., 2015; Mugwanya et al., 2016). Studies also compared the use of daily and intermittent PrEP (Molina et al., 2015), as well as immediate and delayed initiation (McCormack et al., 2016 – not included in the primary meta-analyses but considered in additional analyses below).

The revised Cochrane risk-of-bias tool for randomised trials (RoB 2) was used to assess risk of bias in RCTs [1]. The tool covers several domains of bias: selection bias, performance bias, detection bias, attrition bias, and reporting bias. A series of 'signalling questions' is used to elicit information on trials relevant for assessing bias. These questions can be answered with 'yes', 'probably yes', 'probably no', 'no', and 'no information'. Based on these answers, an algorithm determines a judgement of 'low' or 'high' risk of bias or 'some concerns'. Details on RoB 2 and the signalling questions can be found elsewhere (https://drive.google.com/file/d/19R9savfPdCHC8XLz2iiMvL_71lPJERWK/view). The risk of bias assessment was implemented in the Microsoft Excel-based RoB 2 tool (https://drive.google.com/file/d/1KSFASeBJP8FjBMpEbNlDiYxp4CKuOZgM/view). Information for the risk of bias assessment was drawn from the included articles, including any supplementary material, published protocols for the studies covered by the articles were possible, and non-commercial clinical trial registries such as ClinicalTrials.gov.

The results of the RoB assessment for 18 articles on 12 RCTs in Figures S1 and S2. 17 articles present intention-to-treat (ITT) type analyses; one article presents per-protocol analyses. Overall, risks of bias across articles tended to be low or moderate. Some concerns of biases were found for 14 out of 17 articles with ITT analyses (76.5%), but these concerns tended to be minor. 15 articles with ITT analyses (88.2%) had low risks of biases from the randomisation process and from potential deviations from intended interventions. This was because participants were individually randomised with double-blinding, which was relatively easy to implement due to the available of an adequate placebo for TDF or TDF-FTC. The most common area of concern of bias was due to missing outcome data (76.5% of studies with ITT analyses). Most articles reported some missingness of data, particularly due to loss-to-follow-up. It is plausible that missingness of data could depend on the intervention as those taking PrEP could be more likely to discontinue studies compared to those taking placebos. However, for most studies, loss-to-follow-up and reasons for discontinuation of the study were similar across study groups, suggesting that missingness of data did probably not depend on the trial group membership.

For one article with ITT analyses (5.9%), there were some concerns arising from possible bias in the measurement of the outcome. This article reported on the PROUD study [2]. Unlike all other included studies, this was an open-label study, so outcome assessors were not blinded. However, while in theory creatinine levels could have been evaluated different for different trial groups, this seems unlikely given that the evaluation of serum creatinine and estimation of GFR did not require any judgement from the assessors.

In eight articles with ITT analyses (47.1%), some concerns arose from the selection of the reported results. This was often due to lack of details on the measurement and analysis of creatinine and other outcomes of interest, which were not the primary outcomes in most studies. Particularly, articles that reported on analyses of trials for which main results were reported elsewhere – including for the Partner PrEP trial [3, 4], ANRS-IPERGAY trial [5], and Bangkok Tenofovir trial [6] – concerns arise given that these secondary analyses were not covered in the pre-published study protocols of the trials. However, risks of biases arising from this lack of pre-published protocols is limited given that the outcomes of interest were often determined with well-established methods.

One article reporting on a sub-study of the iPrEx trial [7] was classified as having a high risk of bias from missing outcome data. The article provides very limited information on the completeness of data (unlike the main article on the trial results [8]), so potential for biases cannot be ruled out, although this only affects the results of the sub-study reported in the article, not the results involving all original trial participants.

One article implemented a per-protocol analysis of the Partners PrEP study [4] to evaluate changes in eGFR among study participants who were not off the study drug for 4 or more weeks (thus evaluating eGFR among study participants considered to be more adherent to the intervention). Some concerns arise due to the exclusion

of study participants not adherent to the study drug (which could be due to any reason, including protocol required safety hold). However, the methods were most likely appropriate and the article implemented an intention-to-treat sensitivity analysis (and the main article on the study implemented an intention-to-treat analysis [9]), so risks of bias are limited.

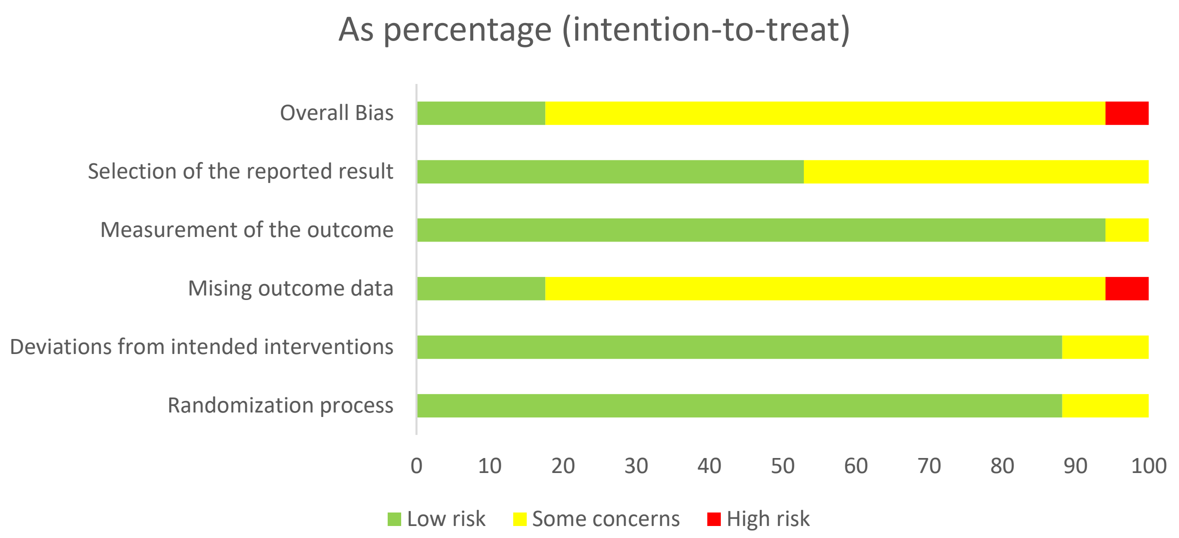

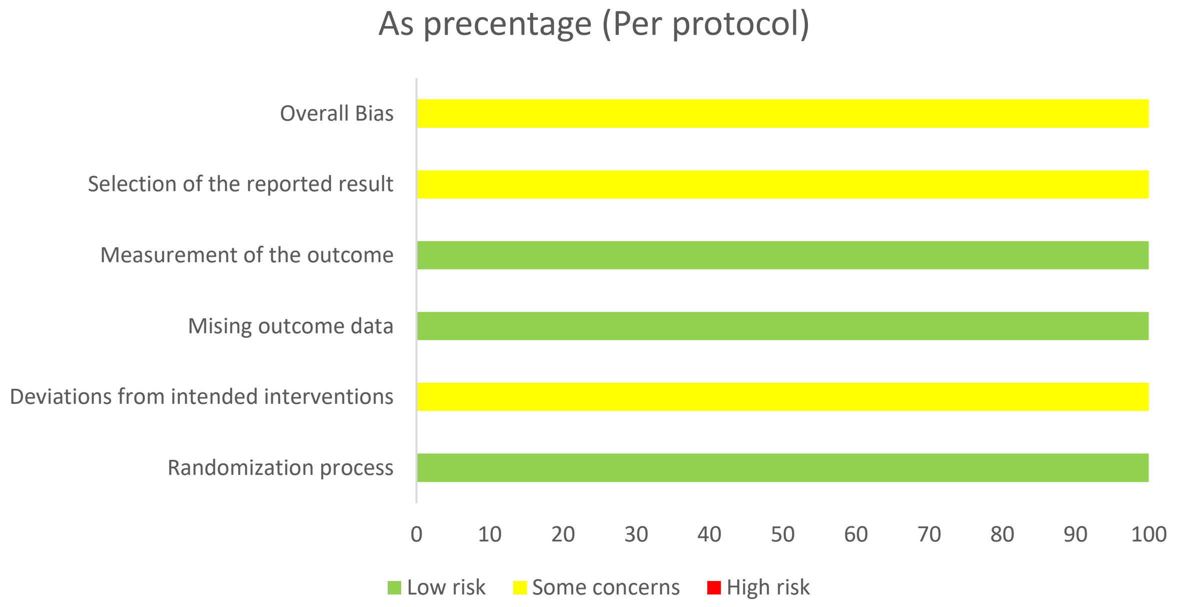

Figure S1: Summary of risk of bias assessment of all included studies. Top: Studies with intention-to-treat analyses (17 articles); bottom: studies with per-protocol analyses (1 article).

ID	Study	Randomization process	Deviations from intended interve	Missing outcome data	Measurement of the outcome	Selection of the reported result	Overall
**Studies with intention-to-treat analyses**							
1	Peterson et al. 2007 West African Safety Study	+	+	?	+	?	!
2	Grant et al. 2010 iPrEx	+	+	?	+	+	!
3	Mutua et al. 2012 IAVI-Kenya	+	+	+	+	+	+
4	Beaten et al. 2012 Partners PrEP	+	+	+	+	?	+
5	Thigpen et al. 2012 TDF2	+	+	?	+	+	!
6	Van Damme et al. 2012 FEM-PrEP	+	?	?	+	+	!
7	Choopanya et al. 2013 Bangkok Tenofovir	+	+	?	+	?	!
8	Grohskopf et al. 2013 US Safety	?	+	?	+	+	!
9	Kibengo et al. 2013IAVI-Uganda	+	+	+	+	+	+
10	Solomon et al. 2014 iPrEx study	+	+	–	+	+	–
11	Martin et al. 2014 Bangkok Tenofovir	+	+	?	+	?	!
12	Mandala et al. 2014 FEM-PrEP	+	+	?	+	+	!
14	Marrazzo et al. 2015 VOICE trial	+	+	?	+	?	!
15	Molina et al. 2015 ANRS-IPERGAY	+	+	?	+	+	!
16	McCormack et al. 2016 PROUD	+	?	?	?	?	!
17	Mugwanya et al. 2016 Partners PrEP	?	+	?	+	?	!
18	Liegeon et al. 2020 ANRS-IPERGAY	+	+	?	+	?	!
**Studies with per-protocol analyses**							
13	Mugwanya et al. 2015 Partner PrEP	+	?	+	+	?	!

+ Low risk

? Some concerns

– High risk

Figure S2: Risk of bias in individual studies.

Systematic literature review and meta-analysis: Meta-analyses using different methods for zero cell studies

The results presented in the main article used a continuity correction of 0.5 for studies with zero events in either study arm. Studies with zero events in both study arms were excluded. In Figure S3, results are presented when using the Peto method to handle zero event studies. The pooled odds ratio (OR) for grade 1+ events was 1.52 (95% confidence interval=1.26-1.84) and 2.04 (0.83-5.02) for grade 2+ events.

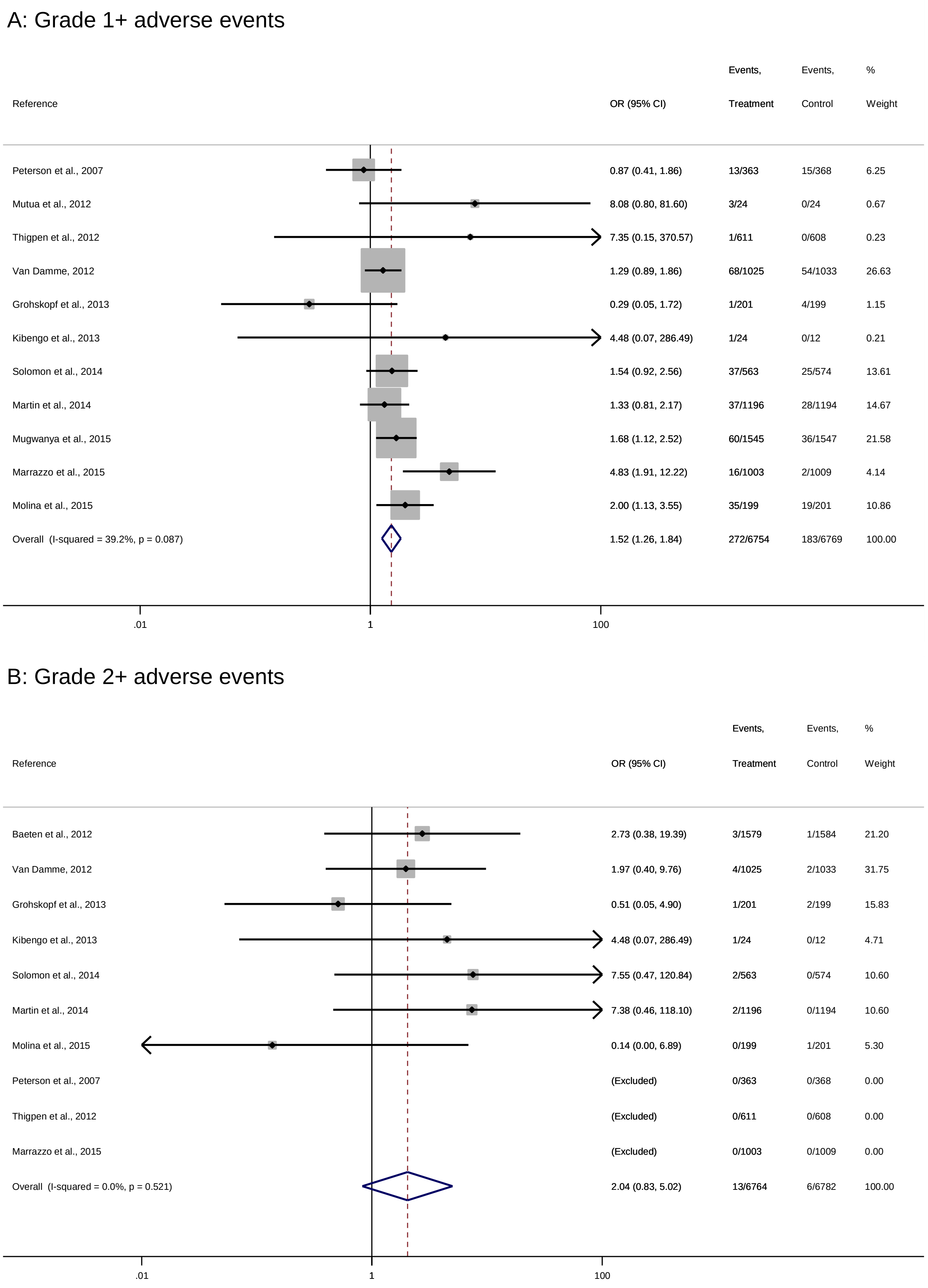

Figure S3: Meta-analysis of grade 1+ kidney-related adverse events (A) and grade 2+ kidney-related adverse events (B) in published randomised controlled trials on oral pre-exposure prophylaxis (PrEP), using the Peto method.

Systematic literature review and meta-analysis: Meta-analyses including two additional RCTs

The primary meta-analyses presented in the main article included data for 11 RCTs. Two additional RCTs were identified (McCormack et al., PROUD study; Hosek et al, PrEPare – ATN082 study), which were not included in the primary meta-analyses. The PROUD trial reported 3 kidney-related adverse events among 273 participants receiving TDF/FTC (0/267 in control group). The trial was not included in our meta-analyses as it did not provide sufficient information on the grading of the kidney-related adverse events, although the study noted that the grading was assessed by the participant's clinician and the serum creatinine elevation in one participant was considered "moderate" while two others were "mild". The study did also not compare outcomes to a placebo but to TDF/FTC after a deferral period of one year. The PrEPare - ATN 082 trial reported two instances of grade 3 creatinine clearances decreases in one participant (out of 20) (0/19 in the placebo arm). Again, insufficient information was provided on how kidney-related adverse events were graded. The study noted that the participants baseline creatinine clearance was 180ml/min and never declined below 110ml/min. This would mean there was at most a 39% decline in creatinine clearance, which would not be classified a grade 3 adverse events as per definitions of the National Institutes of Health, Division of AIDS, used in our study.

We conducted a sensitivity analysis in which the included PROUD and PrEPare - ATN 082 trials. We assumed that the kidney-related adverse events in the trials corresponded to the definitions of the National Institutes of Health, Division of AIDS, used in our study. This means 4 grade 1+ adverse events were added to the meta-analysis (3 from PROUD and 1 from PrEPare – ATN 082) and 2 grade 2+ events (1 from PROUD and 1 from PrEPare – ATN 082). The individual in the PrEPare trial was only counted once for this analysis. Using the same methodology as for the primary meta-analyses (continuity correction of 0.5 for studies with zero events), for grade 1+ adverse events, the pooled risk is 1.53 (95% CI: 1.20-1.94) and for grade 2+ adverse events, the pooled risk is 1.89 (95% CI: 0.79-4.52) (Figure S4). This is similar to the pooled risks reported in the main article without these studies (grade 1+: OR=1.49, 95% CI=1.22-1.81; grade 2+ OR=1.75, 95% CI=0.68-4.49). Results of the sensitivity analysis were also similar when using the Peto method (grade 1+: OR=1.54, 95% CI=1.28-1.86; grade 2+ OR=2.30, 95% CI=0.97-5.42) (not shown).

A: Grade 1+ adverse events

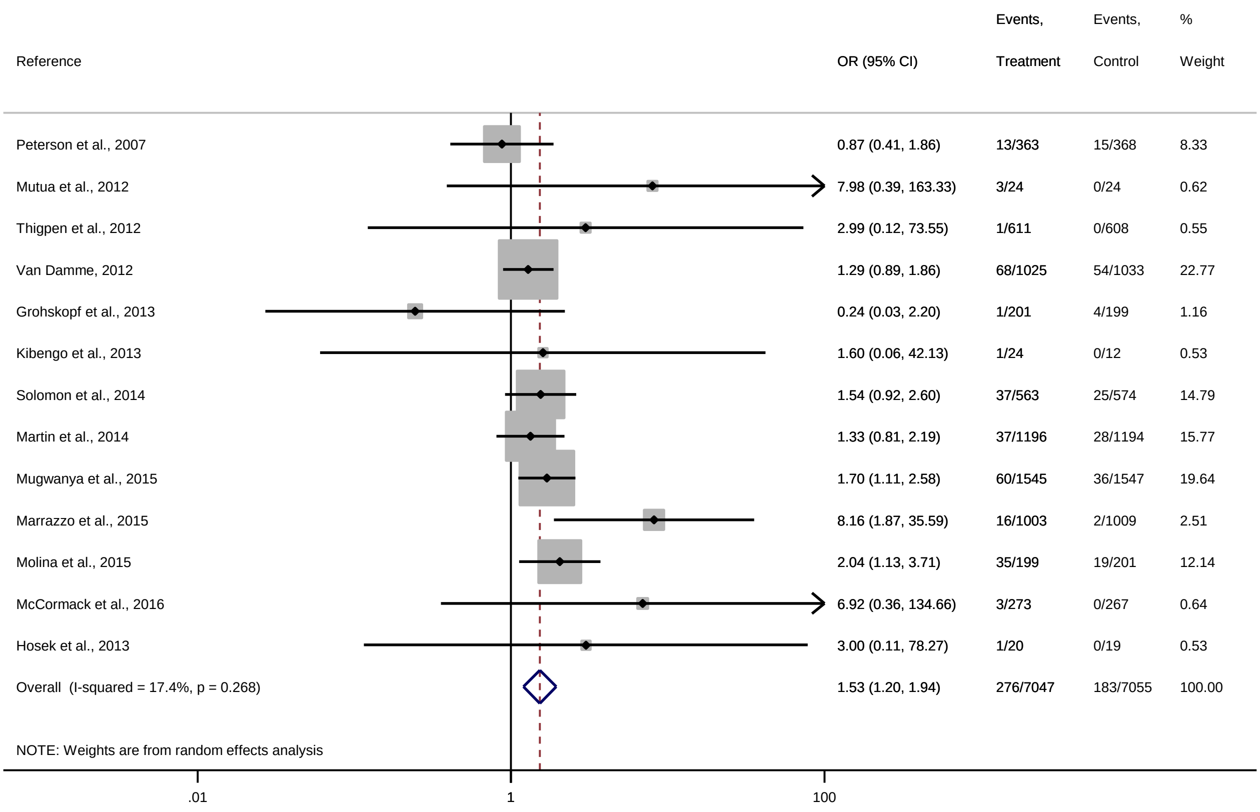

B: Grade 2+ adverse events

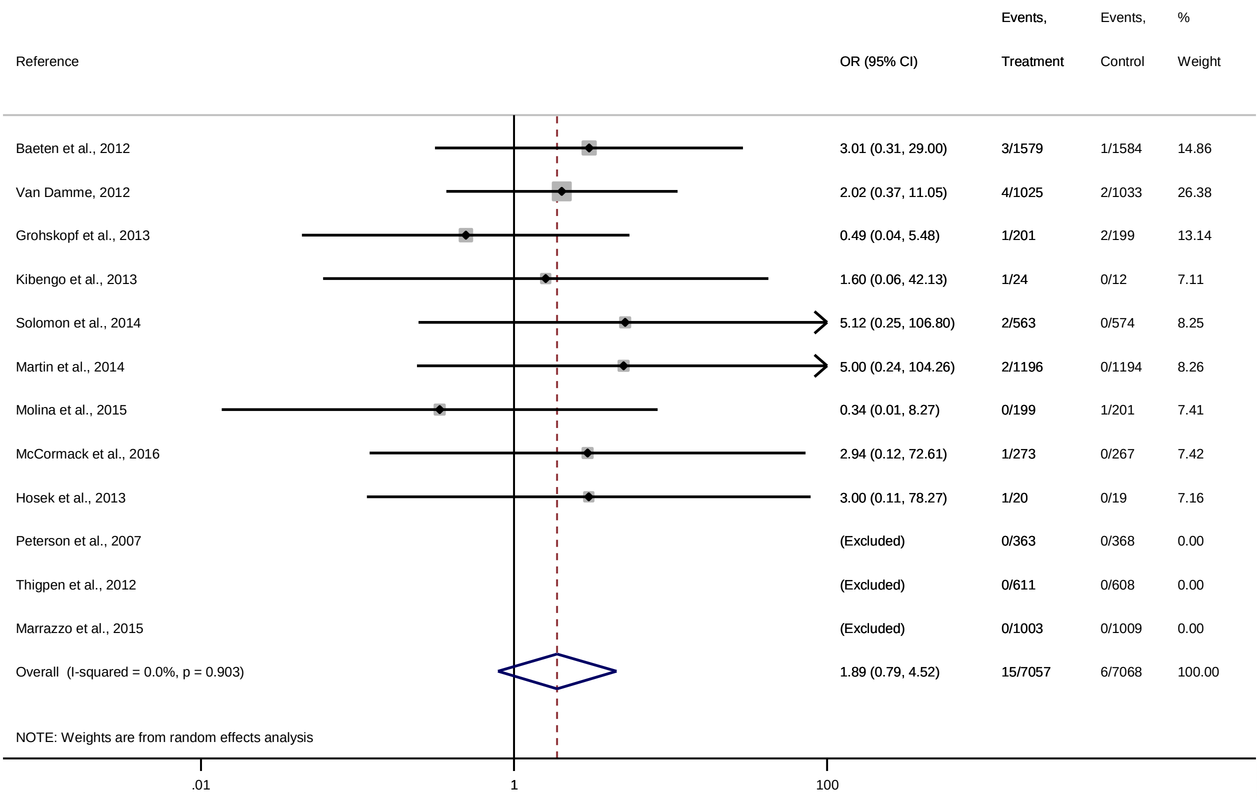

Figure S4: Meta-analysis of grade 1+ kidney-related adverse events (A) and grade 2+ kidney-related adverse events (B) in published randomised controlled trials on oral pre-exposure prophylaxis (PrEP), including two additional RCTs (McCormack et al. and Hosek et al.).

Individual patient data meta-analysis: Details on collected data

All programmes, projects, and studies that contributed data to this study were provided with a standardised form of requested data. Collected data included background information on individuals screened for PrEP initiation, baseline creatinine measurement, and creatinine monitoring data at each follow-up visit after initiation. Table S2 lists all requested variables. Not all of these data were provided by all contributors.

Table S2: Study and individual PrEP user data requested from all contributing partners

Variable
**Study data**
• Country(ies) studied
• Study design
• Study Start date
• Study End date
• Data sources (select all options that apply)
• Study sample size
• Number of HIV-negative people screened for creatinine
**Individual PrEP user data**
• Month and year of birth
• Gender identity of participant/PrEP user
• Race/ethnicity of participant/PrEP user as appropriate to national context
• Weight of PrEP user (*lean body weight if possible*)
• height of PrEP user
• Body mass index (*if known, and height or weight not available*)
• Priority population if known
• Known comorbidities *before* starting PrEP
• Known medication use
• Use of steroids and/or protein supplements
• Known family member with history of kidney disease
• PrEP prescription date (provide variable for each refill date 1, 2, 3, …, ##)
• Date of # creatinine test provide variable for each creatinine test date 1, 2, 3, …, ##)
• Serum creatinine test result for # test
• Estimated creatinine clearance for # test (either from laboratory or Cockcroft–Gault equation)
• Auto-generated creatinine clearance based on serum creatinine, age, weight and sex
• Date of # adverse event (provide variable for each adverse event date 1, 2, 3, …, ##)
• Adverse event #
• Pregnant or breastfeeding woman at PrEP prescription date # (provide variable for each date 1, 2, 3, …, ##)
• PrEP discontinuation due to adverse event
• Date PrEP discontinued
• PrEP restarted after discontinuation
• Adverse events *after restarting* PrEP
• PrEP user in care or lost to follow up
• Date PrEP user exited follow-up either due to discontinuation or loss to follow up
• Any additional notes on client if abnormal creatinine results

Individual patient data meta-analysis: Baseline estimated creatinine clearance

Table S3 presents the distribution of estimated creatinine clearance stages by age, gender, and comorbidities of all individuals included in the baseline analysis of the global data set from 15 countries.

Table S3: Estimated creatinine clearance among individuals screened for PrEP initiation by age, gender, and comorbidities in 15 countries.

		Estimated creatinine clearance stage (%)		
	**n**	**≥90ml/min**	**<90-60ml/min**	**<60ml/min**
**Age (years) (N=18629)**				
15-19	1156	95.3	4.58	0.09
20-24	3631	88.5	11.5	0.03
25-29	4253	83.5	16.3	0.21
30-39	5751	74.8	24.8	0.37
40-49	2584	64.6	34.7	0.70
50+	1254	48.5	49.7	1.83
**Gender (N=18674)**				
Cis male	14194	75.0	24.7	0.32
Cis female	4023	85.7	13.4	0.85
Trans male	35	85.7	14.3	0.00
Trans female	394	81.7	18.3	0.00
Non-binary	28	96.4	3.57	0.00
**Comorbidities (N=776)**				
Diabetes mellitus	23	82.6	8.70	8.70
Dyslipidaemia	7	57.1	42.9	0.00
Hypertension	80	71.3	27.5	1.25

For each age group, gender, and comorbidity, numbers represent the sample sizes for these categories (n) as well as the percentages of individuals in these categories by different estimated creatinine clearance stages. The total sample sizes of individuals with data for these characteristics are indicated (N). Numbers may not add up due to rounding.

Individual patient data meta-analysis: Risks of experiencing a clinically significant decline in estimated creatinine clearance after PrEP initiation

Table S4 presents the incidence and risks of a decline in estimated creatinine clearance to <60ml/min after PrEP initiation for the total data and for low- and middle-income data.

Table S4: Risk of experiencing a clinically significant decline in estimated creatinine clearance to <60ml/min after PrEP initiation by PrEP user age, gender, and baseline estimated creatinine clearance stage in 15 countries.

	All data					Only LMICs				
	**n/N**	**%**	**IR (per 100 pyrs)**	**aHR**	**(95% CI)**	**n/N**	**%**	**IR (per 100 pyrs)**	**aHR**	**(95% CI)**
**Age (years)**										
15-19	2/742	0.27	0.50	1 (reference)		2/649	0.31	0.64	1 (reference)	
20-24	15/2390	0.63	0.79	1.28	(0.29-5.75)	14/1638	0.85	1.25	1.41	(0.31-6.44)
25-29	34/3198	1.06	1.19	1.57	(0.37-6.78)	26/1777	1.46	1.96	1.57	(0.35-6.95)
30-39	101/4672	2.16	2.07	1.63	(0.38-6.91)	70/2182	3.21	3.93	1.49	(0.34-6.49)
40-49	103/2243	4.59	3.84	2.65	(0.62-11.3)	57/693	8.23	9.96	2.59	(0.59-11.41)
50+	94/1115	8.43	6.79	6.05	(1.41-26.0)	8/183	4.37	6.15	2.88	(0.56-14.91)
**Gender**										
Cis male	211/11131	1.90	1.87	1 (reference)		41/3965	1.03	1.63	1 (reference)	
Cis female	136/2992	4.55	5.19	2.43	(0.98-6.00)	136/2989	4.55	5.20	1.64	(0.64-4.19)
Trans male	0/28	0.00	0.00	NA [1]		0/2	0.00	0.00	NA [1]	
Trans female	2/188	1.06	1.55	1.27	(0.29-5.55)	0/172	0.00	0.00	NA [1]	
Non-binary	0/27	0.00	0.00	NA [1]		0/0	-	0.00	NA [1]	
**Baseline estimated creatinine clearance**										
≥90 ml/min	70/11160	0.63	0.64	1 (reference)		46/5724	0.80	1.10	1 (reference)	
60 - <90 ml/min	254/3151	8.06	8.34	8.49	(6.44-11.2)	112/1365	8.21	11.1	6.55	(4.57-9.39)
<60 ml/min	25/57	43.9	52.6	20.8	(12.8-33.8)	19/41	46.4	63.9	16.1	(9.13-28.36)

For each age, gender, and baseline estimated creatinine clearance category, numbers represent numbers of events and sample sizes (n/N), percentage of events, incidence rate (IR) of events per 100 person-years (pyrs), and adjusted hazard ratios (aHR) with 95% confidence intervals (CI). Results are based on a random effects model with age, gender, and baseline estimated creatinine clearance stage as fixed effects and PrEP study/programme as random effects, with the outcome of experiencing an estimated creatinine clearance of <60ml/min (event). Results are presented for the regression model including all data and for the model restricted to data from low- and middle-income countries (LMICs) (excluding data from Australia, France, and the Netherlands).

[1] No hazard ratio could be estimated due to limited sample size.

Individual patient data meta-analysis: Additional results for data from Eswatini and India

In one data set from Eswatini, 10 out of 53 individuals (20.0%) experienced a clinically significant decline to a creatinine clearance of <60ml/min after PrEP initiation; in the data set from India, 118 out of 646 individuals (18.3%) experienced a clinically significant decline. This is markedly higher than the 1-3% of individuals experiencing a clinically significant decline across all other programmes or studies. Table S5 shows the results of the regression model when excluding individuals from these two data sets.

Table S5: Risk of experiencing a clinically significant decline in creatinine clearance to <60ml/min after PrEP initiation by PrEP user age, gender, and baseline creatinine clearance stage, excluding two data sets with high proportions of individuals experiencing a serve decline.

	n/N	%	aHR	(95% CI)
**Age (years)**				
15-19	1/739	0.14	1 (reference)	
20-24	8/2358	0.34	1.51	(0.19-12.3)
25-29	16/3080	0.52	1.87	(0.24-14.6)
30-39	44/4320	1.02	2.40	(0.32-18.3)
40-49	59/2055	2.87	4.15	(0.54-31.8)
50+	93/1109	8.39	9.07	(1.19-69.5)
**Gender**				
Cis male	206/11094	1.86	1 (reference)	
Cis female	13/2330	0.56	1.12	(0.38-3.32)
Trans male	0/28	0.00	NA [1]	
Trans female	2/188	1.06	1.26	(0.29-5.49)
Non-binary	0/27	0.00	NA [1]	
**Baseline creatinine clearance**				
≥90 ml/min	42/10741	0.39	1 (reference)	
60 - <90 ml/min	170/2895	5.87	9.65	(6.74-13.8)
<60 ml/min	9/33	27.3	34.7	(16.4-73.7)

For each age, gender, and baseline creatinine clearance category, numbers represent numbers of events and sample sizes (n/N), percentage of events, and adjusted hazard ratios (aHR) with 95% confidence intervals (CI). Results are based on a random effects model with age, gender, and baseline creatinine clearance stage as fixed effects and PrEP study/programme as random effects, with the outcome of experiencing a creatinine clearance of <60ml/min (event). Results are presented for the regression model excluding one data set from India and one data set from Eswatini.

[1] No hazard ratio could be estimated due to limited sample size.

References

1. Sterne JAC, Savović J, Page MJ, Elbers RG, Blencowe NS, Boutron I, et al. RoB 2: a revised tool for assessing risk of bias in randomised trials. BMJ. 2019;366:l4898. doi:10.1136/bmj.l4898.
2. McCormack S, Dunn DT, Desai M, Dolling DI, Gafos M, Gilson R, et al. Pre-exposure prophylaxis to prevent the acquisition of HIV-1 infection (PROUD): effectiveness results from the pilot phase of a pragmatic open-label randomised trial. Lancet. 2016;387:53–60. doi:10.1016/S0140-6736(15)00056-2.
3. Mugwanya K, Baeten J, Celum C, Donnell D, Nickolas T, Mugo N, et al. Low Risk of Proximal Tubular Dysfunction Associated With Emtricitabine-Tenofovir Disoproxil Fumarate Preexposure Prophylaxis in Men and Women. J Infect Dis. 2016;214:1050–7. doi:10.1093/infdis/jiw125.
4. Mugwanya KK, Wyatt C, Celum C, Donnell D, Mugo NR, Tappero J, et al. Changes in Glomerular Kidney Function Among HIV-1–Uninfected Men and Women Receiving Emtricitabine–Tenofovir Disoproxil Fumarate Preexposure Prophylaxis: A Randomized Clinical Trial. JAMA Intern Med. 2015;175:246–54. doi:10.1001/jamainternmed.2014.6786.
5. Liegeon G, Antoni G, Pialoux G, Capitant C, Cotte L, Charreau I, et al. Changes in kidney function among men having sex with men starting on demand tenofovir disoproxil fumarate – emtricitabine for HIV pre-exposure prophylaxis. J Int AIDS Soc. 2020;23:e25420. doi:10.1002/jia2.25420.
6. Martin M, Vanichseni S, Suntharasamai P, Sangkum U, Mock PA, Gvetadze RJ, et al. Renal Function of Participants in the Bangkok Tenofovir Study—Thailand, 2005–2012. Clin Infect Dis. 2014;59:716–24. doi:10.1093/cid/ciu355.
7. Solomon MM, Lama JR, Glidden D V, Mulligan K, McMahan V, Liu AY, et al. Changes in renal function associated with oral emtricitabine/tenofovir disoproxil fumarate use for HIV pre-exposure prophylaxis. AIDS. 2014;28:851–9. doi:10.1097/QAD.0000000000000156.
8. Grant RM, Lama JR, Anderson PL, McMahan V, Liu AY, Vargas L, et al. Preexposure Chemoprophylaxis for HIV Prevention in Men Who Have Sex with Men. N Engl J Med. 2010;363:2587–99. doi:10.1056/NEJMoa1011205.
9. Baeten JM, Donnell D, Ndase P, Mugo NR, Campbell JD, Wangisi J, et al. Antiretroviral Prophylaxis for HIV Prevention in Heterosexual Men and Women. N Engl J Med. 2012;367:399–410. doi:10.1056/NEJMoa1108524.
